# Cost-analysis and quality of life after laparoscopic and robotic ventral mesh rectopexy for posterior compartment prolapse: a randomized trial

**DOI:** 10.1007/s10151-019-01991-2

**Published:** 2019-05-08

**Authors:** J. Mäkelä-Kaikkonen, T. Rautio, A. Ohinmaa, S. Koivurova, P. Ohtonen, H. Sintonen, J. Mäkelä

**Affiliations:** 1 Division of Gastroenterology, Department of Surgery, University Hospital of Oulu, PO Box 21, 90029 Oulu, Finland; 2 Center of Surgical Research, Medical Research Center, University of Oulu, Oulu, Finland; 3 School of Public Health, University of Alberta, Edmonton, AB Canada; 4 Department of Obstetrics and Gynecology, University Hospital of Oulu, Oulu, Finland; 5 Department of Public Health, University of Helsinki, Helsinki, Finland

**Keywords:** Ventral mesh rectopexy, Laparoscopic, Robotic, Cost-analysis, Quality of life

## Abstract

**Background:**

The aim of this study was to assess, whether robotic-assistance in ventral mesh rectopexy adds benefit to laparoscopy in terms of health-related quality of life (HRQoL), cost-effectiveness and anatomical and functional outcome.

**Methods:**

A prospective randomized study was conducted on patients who underwent robot-assisted ventral mesh rectopexy (RVMR) or laparoscopic ventral mesh rectopexy (LVMR) for internal or external rectal prolapse at Oulu University Hospital, Finland, recruited in February–May 2012. The primary outcomes were health care costs from the hospital perspective and HRQoL measured by the 15D-instrument. Secondary outcomes included anatomical outcome assessed by pelvic organ prolapse quantification method and functional outcome by symptom questionnaires at 24 months follow-up.

**Results:**

There were 30 females (mean age 62.5 years, SD 11.2), 16 in the RVMR group and 14 in the LVMR group. The surgery-related costs of the RVMR were 1.5 times higher than the cost of the LVMR. At 3 months the changes in HRQoL were ‘much better’ (RVMR) and ‘slightly better’ (LVMR) but declined in both groups at 2 years (RVMR vs. LVMR, *p* > 0.05). The cost-effectiveness was poor at 2 years for both techniques, but if the outcomes were assumed to last for 5 years, it improved significantly. The incremental cost-effectiveness ratio for the RVMR compared to LVMR was €39,982/quality-adjusted life years (QALYs) at 2 years and improved to €16,707/QALYs at 5 years. Posterior wall anatomy was restored similarly in both groups. The subjective satisfaction rate was 87% in the RVMR group and 69% in the LVMR group (*p* = 0.83).

**Conclusions:**

Although more expensive than LVMR in the short term, RVMR is cost-effective in long-term. The minimally invasive VMR improves pelvic floor function, sexual function and restores posterior compartment anatomy. The effect on HRQoL is minor, with no differences between techniques.

## Introduction

External rectal prolapse (ERP) and internal rectal prolapse (IRP) with symptoms of obstructed defecation and/or fecal incontinence are debilitating conditions resulting in impairment of the patients’ quality of life [1]. Laparoscopic ventral mesh rectopexy (LVMR) is proposed as the treatment of choice for ERP and is also increasingly performed to treat symptomatic IRP in selected patients [2–6]. Robot-assisted surgery offers an alternative to laparoscopy in rectopexy operations with its acknowledged technical advantages, which may be of benefit when operating in confined pelvic space [7–9]. Between 2012 and 2015 the largest increase in robot use in colorectal surgery in the United States happened in robotic rectopexy which increased from 15 to 27% with contemporaneous 12% decrease in open rectopexies and overall increase in laparoscopic approaches from 30 to 32% [10].

The role of robot-assisted laparoscopy in treating posterior pelvic floor dysfunction is undetermined, based on current evidence of non-randomized relatively small comparative series of rectopexy operations [11–13]. It is not known, if robotic ventral mesh rectopexy (RVMR) offers improvements in medical care and operative outcomes in the form of better anatomical and functional results, improved health-related quality of life (HRQoL) and a reduced recurrence rate. A systematic review and meta-analysis of 6 studies and 340 patients showed less intraoperative bleeding, lower incidence of postoperative complications and shorter hospital stay for RVMR compared with LVMR, but found no differences in rates of recurrence, conversion or reoperation [14]. Major drawbacks limiting robot-use are increased cost and longer operating times [14]. The cost-effectiveness of robot-use in rectopexy operations has not been evaluated using cost-utility analysis. Health care decision makers need more information from economic evaluations to show whether implementing the new expensive robotic technology is providing extra value for the society. The aim of this study was to evaluate the incremental cost-effectiveness in terms of cost per quality-adjusted life year (QALY) gained of RVMR compared to LVMR using hospital perspective and secondarily, to compare the effect of RVMR and LVMR for posterior compartment procidentia on pelvic floor anatomy and function in the long-term.

## Materials and methods

**Fig. 1:**
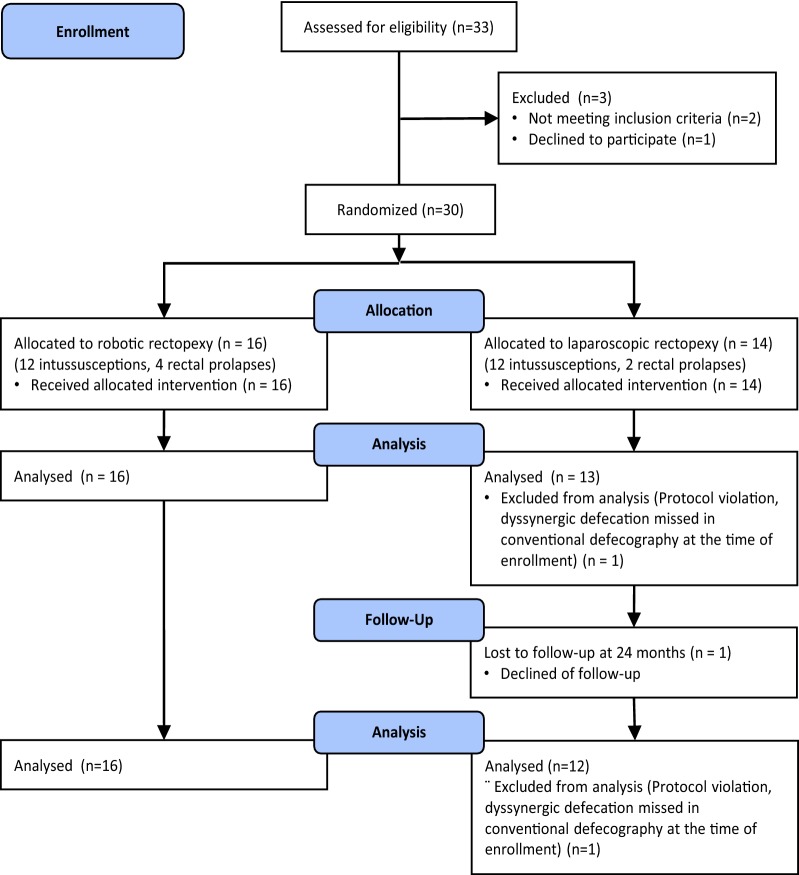
Flow chart of study

This study is a part of a prospective randomized controlled series comparing RVMR and LVMR operations registered in Current Controlled Trials, ISRCTN88884232. The study protocol has been approved by the Ethics Committee of the Oulu University Hospital. Within a concept of pilot study, we aimed to include 30 patients in the trial. Thirty-three consecutive female patients who were diagnosed with ERP or recto-anal IRP with or without entero/rectocele combined with symptoms of obstructive defecation and/or fecal incontinence were recruited to the study between February and May 2012. All patients signed written informed consent. The randomization method is explained in our previous report of short-term results at 3-month follow-up [15]. Allocation and follow-up are displayed in Fig. 1. Preoperative diagnostics included clinical anorectal examination, pelvic examination, colonoscopy, and magnetic resonance (MR)-defecography.

### Surgical technique

The robotic operations were performed by three surgeons and in addition fourth surgeon participated in laparoscopic operations. Our surgical technique follows the protocol described by D’Hoore and Penninckx [3], with slight modifications described earlier [16]. The rectovaginal space was dissected deep to the levator plane with harmonic scalpel. A single polypropylene mesh (Parietex™, size 3 cm × 20 cm; Covidien, Dublin, Ireland) was fixated caudally with two resorbable sutures through the pelvic floor using an endofascial closing device and thereafter anteriorly to the rectum and to the apex of the vagina with 6–7 pairs of non-absorbable sutures. For the suspension to the sacral promontory spiral attachments were used (Pro-Tack™ Fixation Device, Covidien) and peritoneum was closed over the mesh with continuous suture with 15-cm long V-Loc™ (Covidien). The RVMR were performed following the same protocol with Si Da Vinci Surgical System (Intuitive Surgical Inc, Sunnyvale, CA, USA). For the robotic procedures we used side docking and five trocar placements. The patients were blinded to the operative technique.

### Effectiveness measurement

All patients had standardized evaluation preoperatively and at 3- and 24-month follow-up. The HRQoL was measured by the generic preference-based 15D instrument [17]. The HRQoL of patients were compared with that of a sample of general Finnish female population derived from a representative population survey, Health 2011 [33]. The disease-specific instruments included the Finnish translations of the Pelvic Floor Distress Inventory (PFDI-20), and the Pelvic Floor Impact Questionnaire 7 (PFIQ-7) [18], the Wexner score for anal incontinence [19] and the Obstructed Defecation Syndrome score (ODS score) [20]. POP-Q measurements (Pelvic Organ Prolapse Quantification) [21] were obtained in supine lithotomy position during maximal Valsalva.

### Cost-analysis

The cost-analysis includes the costs of surgical intervention, hospitalization and the cost of reoperations using the same method as original surgery, and other major complications. The costs of surgical intervention includes the capital costs of robot- and laparoscopy instrument use and the operating time of the personnel measured in minutes and multiplied by personnel’s salary including benefits from the hospital accounting. The capital cost of robot and laparoscopy equipment was divided over 10 years using 2% hospital specific interest rate and calculated per procedure. The cost of postoperative hospitalization including medication costs were extracted from hospital records. Considering the small study sample, we used reoperation rates from 2007 to 2013 to estimate re-surgery rate for RVMR (2.2%) and LVMR (11.6%). The lost productivity due to sick leave and possible additional postoperative health and social services were surveyed during the follow-up visits. All costs are reported as Euros and were originally valued at the 2012 price level and then inflated to the 2017 price level using the price index of public health care [22]. The costing details are in the footnote of Table 4.

### Economic analysis

Economic analysis was done using incremental cost-effectiveness analysis [23]. The HRQoL outcomes were assessed using the 15D instrument that provides a preference-based single index value for each measurement, before operation, and 3 and 24 months postoperatively. Quality-adjusted life years (QALYs) gained after surgery were estimated using individual level 15D scores at baseline to represent no surgery option and comparing it to estimated area below curve during 2-year follow-up after surgery and estimating the difference between these two lines. In the final analysis incremental cost per incremental QALY ratio for the RVMR compared to LVMR was estimated for 2 years as the base case, and for 5 years assuming constant annual QALY gain and no additional incremental costs (same healthcare services).

We used 3% discount rate to calculate 2 and 5 years QALY results. The number of robotic surgeries (284/year) was based on our hospital 2015–2017 surgery numbers. The main analysis was done using the operating hospital perspective, since the pre- and postoperative health care services outside the hospital were estimated to be the same.

Sensitivity analysis was done using one way and multiway sensitivity analysis for key cost variables, like operation time, equipment lifetime of 7 years, and number of reoperations, 200 operations/year, and 0% discount rate for QALY.

### Statistical analysis

Statistical analysis was performed using SPSS for Windows (released 2015, version 23.0. IBM, Armonk, NY, USA). Differences between the study groups were analyzed using Student’s *t* test for continuous data and Chi-squared test or Fisher’s exact test for categorical data. If both preoperative and postoperative values were calculated, then first the difference between the preoperative and postoperative value was calculated (change) and the between group comparison of the changes was compared using a *t* test (continuous variables), Mann–Whitney test (semi-continuous variables) or Fisher’s exact test (categorical variables). Variables with more than two repeated measurements were analyzed using Linear Mixed Model (LMM) assuming random subject effect. The covariance pattern for LMM was chosen according to Akaike’s information criteria. *p* values reported for LMM are p-time, indicating change over time, p-group indicating average between group difference, and p-time × group indicating interaction between time and group. A *p* value of less than < 0.05 was considered statistically significant.

## Results

### Clinical effectiveness

**Fig. 2:**
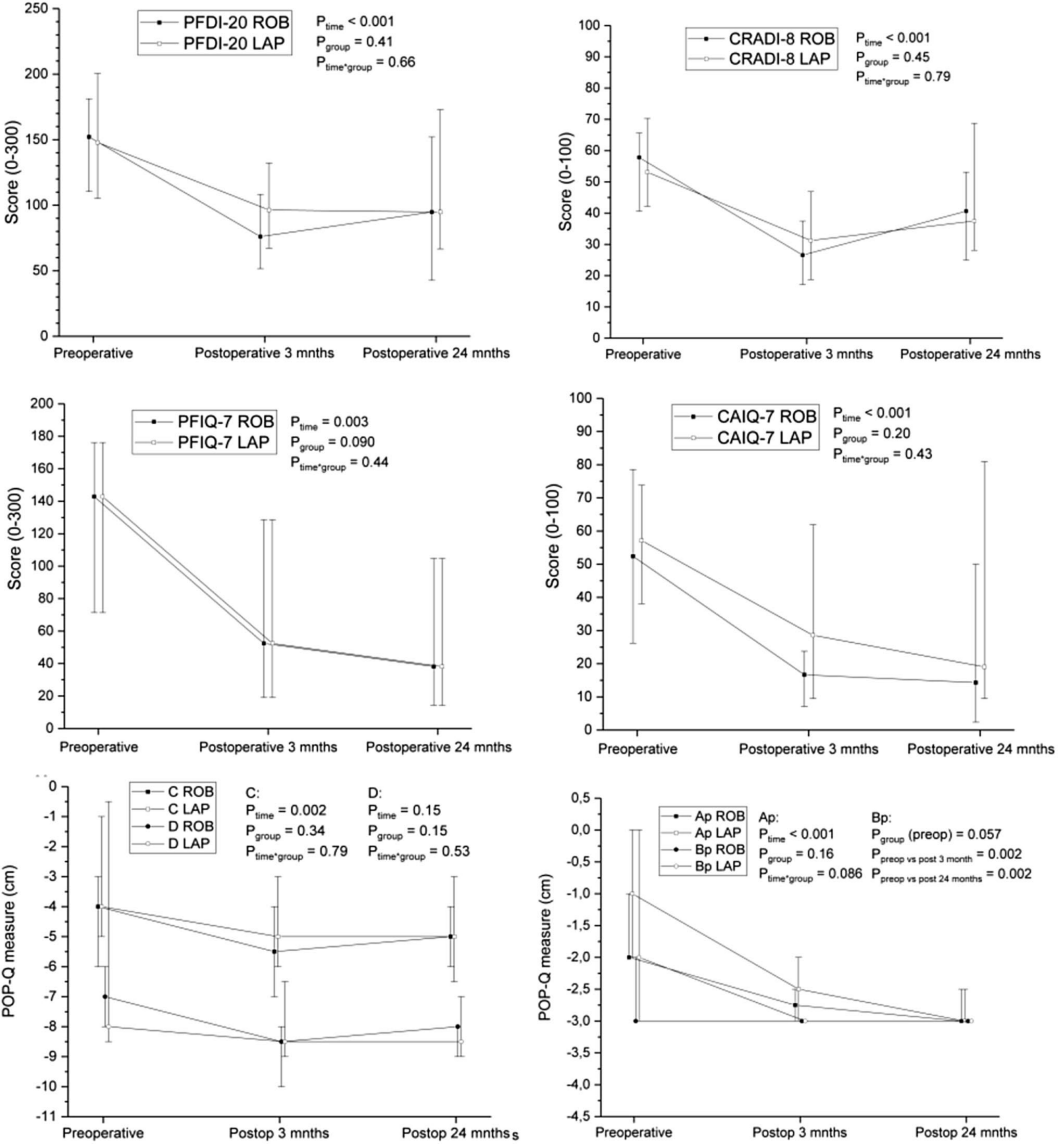
The comparison of changes in median symptom scores, symptom-specific QoL scores and posterior wall POP-Q measurements for the RVMR and LVMR groups. *PFDI-20* Pelvic Floor Distress Inventory, *CRADI-8* Colorectal-Anal Distress Inventory, *PFIQ-7* Pelvic Floor Impact Questionnaire, *CAIQ-7* Colorectal-anal Impact Questionnaire, *POP-Q* pelvic organ quantification, all measures taken at maximum strain with reference to the level of hymen: Ap, point 3 cm above hymen in the posterior wall, Bp the most protruding point of the posterior wall. Data presented as medians with 25th and 75th percentiles

From 30 female patients (62.5 years, SD 11.2) operated on, 16 patients (4 ERP and 12 IRP) had RVMR and 14 patients (2 ERP and 12 IRP) LVMR. Table 1 summarizes the anatomical, functional and condition-specific quality of life outcomes at baseline and at 24-month follow-up. An improvement was seen in pelvic floor distress inventory scores (PFDI) and specifically in the colorectal-anal (CRADI) and pelvic organ prolapse (POPDI) subscales. An overall improvement in obstructed defecation was achieved in 84.6% of RVMR patients and in 41.7% of LVMR patients. In total, 48.1% of patients reported fecal incontinence before surgery (RVMR 50%, LVMR 46.2%) with no significant improvement in Wexner scores at the follow up. Table 2 summarizes the POP-Q stage changes after the operation. All patients in both study groups had maintained the posterior wall stage ≤ 1. No differences were found either in the anatomical or functional parameters between the RVMR and LVMR groups (Fig. 2). Prior to the 24-month follow-up, one patient in LVMR group had been treated with RVMR for ERP recurrence, thereby giving an 8% failure rate for the LVMR group compared to no reoperations in the RVMR group.

**Table 1:** Symptom scores, quality of life measures and POP-Q measurements before surgery and at 24 months after RVMR and LVMR

	Before surgery				24 months after surgery				Mean difference						RVMR vs. LVMR
	RVMR *n *= 16		LVMR *n *= 13		RVMR *n *= 15		LVMR *n *= 13		RVMR			LVMR			
	Md	25th–75th percentile	Md	25th–75th percentile	Md	25th–75th percentile	Md	25th–75th percentile	Mean	SD	*p* value	Mean	SD	*p* value	*p* value
Symptom scores															
Wexner	9.5^a^	1.5 to 14.5	7	3.5 to 15.5	10	3 to 14	4	3.0 to 13.5	− 0.5	4.6	0.72	− 1.9	0.8	0.385	0.55
ODS	18^a^	16.8 to 21.8	21^b^	17.3 to 25.3	14	5 to 14	19	6.5 to 23.0	− 7.6	7.4	0.002	− 3.4	10.2	0.247	0.37
CRADI-8	58	39 to 67	53^b^	41 to 73	41	25 to 56	38	27 to 70	− 16.0	19.0	0.005	− 9.1	24.3	0.220	0.43
POPDI-6	52	38 to 63	50^c^	25 to 63	17	8 to 33	25	15 to 60	− 25.0	29.7	0.005	− 14.4	29.9	0.141	0.37
UDI-6	42	19 to 54	50	31 to 68	29	8 to 79	42	19 to 67	3.1	33.5	0.729	− 6.9	27.3	0.397	0.41
PFDI-20	152	109 to 182	148^c^	100 to 218	94.8	40 to 152	95	65 to 181	− 38.0	71.5	0.059	− 23.9	61.7	0.229	0.60
Quality of life measures															
CAIQ-7	52	25 to 80	57^b^	38 to 75	14.3	0 to 52	19.0	7 to 81	− 25.0	30.1	0.007	− 19.6	44.4	0.154	0.74
POPIQ-7	7	0 to 23	52^c^	0 to 63	0	0 to 5	0	0 to 20	− 2.5	30.3	0.750	− 39.2	31.7	0.004	0.10
UIQ-7	29	6 to 56	38	24 to 48	28.6	0 to 62	19.0	5 to 81	− 2.9	39.4	0.783	− 7.1	25.8	0.380	0.80
PFIQ-7	98	48 to 145	152^c^	63 to 177	28.6	10 to 129	48	17 to 160	− 30.2	89.0	0.210	− 71.7	92.2	0.036	0.79
15D score	0.848	0.703 to 0.911	0.839	0.755 to 0.875	0.876	0.876 to 0.944	0.832	0.763 to 0.846	0.011	0.12	0.726	− 0.003	0.43	0.804	0.47
POP-Q measurements															
Ap	− 2	−2 to − 1	− 1	− 2 to 0.8	− 3	− 3 to − 2.5	− 3	− 3 to − 2.5	− 1.2	1.0	0.000	− 2.2	1.8	0.001	0.07
Bp	− 3	− 3 to − 2	− 2	− 3 to 0.5	− 3	− 3 to − 3	− 3	− 3 to − 3	− 0.5	1.0	0.056	− 2.1	2.5	0.011	0.054
C	− 4	− 6 to − 3	− 4	− 6 to 0	− 5	− 6 to − 4	− 5	− 6.8 to − 2.5	− 0.8	1.9	0.136	− 1.3	3.0	0.130	0.55
D	− 7^d^	− 8.3 to − 7.8	− 8^e^	− 9 to 3	− 8^d^	− 9.3 to − 8	− 8.5^e^	− 9 to − 7	− 1.4	1.6	0.024	− 4.6	8.0	0.183	0.81
Aa	− 1	− 2 to 0	− 1	− 2 to 1	− 1.5	− 2 to 0	0	− 2 to 1	− 0.1	1.2	0.827	0.3	1.5	0.423	0.43
Ba	− 1	− 3 to 0	0	− 3 to 1	− 2	− 3 to − 1	− 0.5	− 3 to 0.3	− 0.4	1.4	0.238	− 0.6	1.7	0.256	0.81

Student’s *t* test, statistically significant at *p *= 0.05 level (two-tailed)

Data presented as medians with 25th and 75th percentiles

POP-Q pelvic organ quantification, all measures taken at maximum strain with reference to the level of hymen: Ap point 3 cm above hymen in the posterior wall, Bp the most protruding point of the posterior wall, C cervix or hysterectomy scar, D posterior fornix of vagina, Aa point 3 cm above hymen in the anterior wall, Ba the most protruding point of the anterior wall

*CRADI-8* Colorectal-Anal Distress Inventory, *CAIQ-7* Colorectal-anal Impact, *POPDI-6* Pelvic Organ Prolapse Distress Inventory, *POPIQ-7* Pelvic Organ Prolapse Impact, *UDI-6* Urinary Distress Inventory, *UIQ-7* Urinary Impact, *PFDI-20* Pelvic Floor Distress Inventory, *PFIQ-7* Pelvic Floor Impact Questionnaire

Number of patients ^a^*n *= 14, ^b^*n *= 12, ^c^*n *= 11, ^d^*n *= 10, ^e^*n *= 7

**Table 2:** POP-Q stage changes after RVMR and LVMR surgery

Compartment	Before surgery	3 months	24 months	*p* value^a^ 3 months	*p* value^b^ 3 months	*p* value^a^ 24 months	*p* value^b^ 24 months
Posterior wall stage (0/1/2/3)							
All	4/10/13/2	14/14/1/0	16/12/0/0	0.186	< 0.001	0.667	< 0.001
Robotic	2/7/7/0	8/7/1/0	8/7/0/0		0.006		0.004
Laparoscopic	2/3/6/2	6/7/0/0	8/5/0/0		0.007		0.001
Apex stage (0/1/2/3)							
All	9/14/4/2	13/14/2/0	11/16/1/0	0.119	0.020	0.841	0.156
Robotic	5/10/1/0	6/10/0/0	6/9/0/0		1.000		1.000
Laparoscopic	4/4/3/2	7/4/2/0	5/7/1/0		0.031		0.180
Anterior wall stage (0/1/2/3)							
All	3/7/16/3	2/12/15/0	2/11/15/0	0.640	0.109	0.396	0.273
Robotic	2/3/11/0	1/8/7/0	2/6/7/0		0.375		0.375
Laparoscopic	1/4/5/3	1/4/8/0	0/5/8/0		0.375		0.750

^a^Robot-assisted and laparoscopic groups tested using the Mann–Whitney *U* test

^b^Wilcoxon signed-rank test, based on positive ranks

### Health-related quality of life

**Fig. 3:**
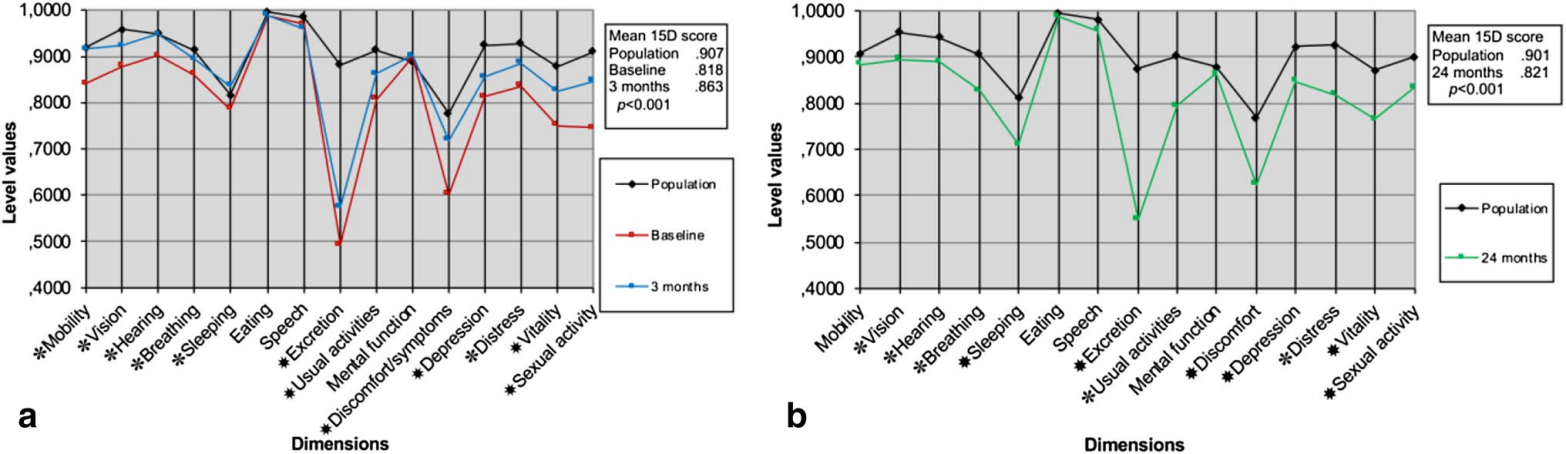
HRQoL profiles of study population at baseline and at 3 and 24 months after VMR surgery vs. general population. Age and gender-adjusted 15D profiles of the patients with symptomatic posterior compartment procidentia in comparison to general female population [H2000 (**a**), H2011, (**b**)]. ^✽^*p* < 0.05, ^✸^*p* < 0.001

Figures 2 and 3 show the mean 15D scores at different time points. At baseline the study population had significantly reduced scores on the dimensions of excretion and discomfort/symptoms compared to general population (Fig. 3a, b). At 3 months the dimensions of excretion, sexual activity and discomfort/symptoms were somewhat improved, but by 24 months the HRQoL scores were back to the original or slightly higher levels (*p* = NS) than at baseline except for sexual activity that remained at a higher level (*p* = NS). At 3 months, the mean 15D score had improved from baseline + 0.062 for the RVMR group and + 0.023 for the LVMR group, both exceeding the minimal important change (MIC) of ± 0.015. At 24 months both had declined, the RVMR mean score being + 0.011 and LVMR mean score being − 0.003 from the baseline. The incremental QALYs gained over 2 years in favor to RVMR group was 0.0435 (SE 0.480) using 3% discounting and 0.0454 (SE 0.0483) with no discounting (Table 3). At 24 months 87% and 69% of patients were satisfied, and 6% and 31% were dissatisfied with the end result in the RVMR and LVMR groups (*p* = NS), respectively.

**Table 3:** HRQoL in the 2-year follow-up and incremental QALYs gained

Surgery	Baseline Mean (95% CI)	3 months Mean (95% CI)	24 months Mean (95% CI)	Incremental QALYs	QALYs gained in 2 years
Robotic surgery	0.816 (0.755, 0.876)	0.878 (0.815, 0.941)	0.822 (0.737, 0.906)	0.0671 (SE 0.038, SD 0.152)	0.0454 (SE 0.0483)^a^
Laparoscopic surgery	0.822 (0.788, 0.856)	0.845 (0.796, 0.895)	0.820 (0.781, 0.860)	0.0217 (SE 0.026, SD 0.094)	

^a^QALYs gained using 3% discounting was 0.0435 for 2 years and 0.104 for 5 years

### Cost-effectiveness analysis

The details of the surgery-related hospital costs are shown in Table 4. The RVMR was about 1.5 times more expensive than LVMR, the inflation corrected costs being €5211 for robotic and €3472 for laparoscopic operations (difference: €1739 at the 2017 price level). The costs of the operation theatre and personnel and duration of hospitalization did not differ significantly. After discharge patients in both groups had only few additional physician visits and also the sick leaves did not differ statistically significantly (RVMR 32 days vs. LVMR 36 days, *p* = 0.61) and they were excluded from the additional analysis. The incremental cost-effectiveness ratio (ICER) for the RVMR compared to LVMR was €39,982/QALY gained at 2 years (Table 5). Due to low QALY gain compared to baseline in the LVMR group, its cost/QALY gained was very high (over €167,744) and about twice the 2-year ratio of the RVMR group (€81,176).

**Table 4:** Costs for the robotic and laparoscopic ventral mesh rectopexy

Cost categories	RVMR	LVMR
Operating theatre cost^a^	92	90
Human resources^b^	373 (76)	376 (62)
Anesthesia/recovery room time		
Instruments and disposables		
Harmonic	–	400
Disposable ports	200	253
Protack	195	195
Mesh	130	130
Stitches	45	45
Other disposables	157.40	130.60
Use of DaVinci		
Intuitive instruments	1472	
Maintenance cost	493	
Amortization		
DaVinci^c^	637	–
Laparoscopic column^d^	–	18.4
Hospitalization^e^	1199	1362.5
Complications (re-surgery)	120.9	406.1
Total cost in 2012 per procedure	5114.3	3407.6
Total in 2017 prices^f^	5211.5	3472.3

^a^Cost for operating room usage: 0.46 €/min

^b^Cost for operating room human resources including two surgeons and one anesthetist (0.615 € per minute per capita) and three nurses (0.21 € per minutes per capita)

^c^Maintenance 140,000 €/year for 284 patient/year

^d^Based on 1,625,000 € investment cost, 10 years use, 284 patient/year and 2% hospital accounting interest

^e^Based on a 47,000 € investment cost, 10 years of use, 284 patients/year and 2% hospital accounting interest

^f^Inflation between 2012 and 2017 was 3.4%, Statistics Finland (2018)

**Table 5:** Incremental cost-effectiveness analysis for 2- and 5-year duration of surgical outcomes with and without QALYs discounted by 3%

Incremental cost/QALY, Euros	2 years 0% discount	2 years 3% discount	5 years 0% discount	5 years 3% discount
Robotic	77,784	81,176	31,076	33,929
Laparoscopic	160,754	167,744	64,183	70,006
Incremental cost/QALY	38,308	39,982	15,310	16,707

In the sensitivity analysis we varied key cost and outcome variables in one-way and multiway analysis. In the cost estimation we tested the impact of using the observed re-surgery rate (8% in LVMR group) which did not have any impact on the ICER. The use of 0% discount rate for QALY reduced the ICER to €38,308/QALY gained. The 5-year ICER assuming no additional surgery related costs and constant QALY gain was €16,707/QALY. Decreasing the annual utilization rate from 284 to 200 patients increased the 2-year ICER to €46,418/QALY and 5-year ICER to €19,397/QALY. A shorter 7-year investment period increased the above estimates about €3000/QALY gained.

## Discussion

The present study provides new data on the HRQoL and cost-effectiveness on robot-assisted ventral mesh rectopexy compared with conventional laparoscopy. Considering the commonly used “willingness to pay threshold” of $50,000 (€43,500), the average gains are poor for both operation methods, but the ICER for RVMR over LVMR (€39,982/QALY) at 2 years is acceptable [24]. However, when we assumed the operative results to last for 5 years, the RVMR technique becomes more cost-effective in terms of ICER (€16,707/QALY) comparing it to the LVMR. The reinforcement to the rectovaginal septum was maintained at follow-up as the clinical assessment of posterior and apical compartments showed reduction of the maximal POP at strain. However, the RVMR or LVMR did not have an impact on generic HRQoL at 24-month follow-up. Despite the trend towards better function and subjective satisfaction in the RVMR group, the differences between RVMR and LVMR were statistically non-significant.

There are no previous full economic evaluations (cost–benefit, cost-effectiveness and cost-utility analyses) comparing RVMR vs. LVMR. In our study the RVMR was 1.5 times more expensive than LVMR, when operation, investment and hospitalization costs were included. In the previous studies in different indications the use of robotics have added between €600–€5359 per procedure to laparoscopic counterpart, but these studies have also great variation in cost-calculations, including, e.g., amortization, impact of annual case-load, and distribution of robotic use between different departments [25, 26]. A cost-analysis by Heemskerk from 2007 showed RVMR to be more expensive than LVMR, which was in part due to increased time-consumption (39 min) in the RVMR [11]. Operative efficiency has been found to be essential in reducing operative cost and achieving profitability and operative time is predicting operative cost more than any other factor [27]. Statistical models have demonstrated that robotic-assisted surgery can achieve cost equivalence to laparoscopic technique, when operative times are reduced below certain levels [28]. In the rectopexy operation time does not seem to play a significant role when both operations are performed by experienced surgeons. In our study, neither operating times (RVMR 125 min vs. LVMR 130 min, *p* = 0.52) nor length of stay (LOS) (RVMR 2.2 days vs. LVMR 2.5 days, *p* = 0.71) differed between the techniques. The increased costs mainly consisted of robotic investment, maintenance and instrument use.

After VMR the postoperative recovery and complications are short term measures whereas anatomic, functional and HRQoL improvements for the patient are long-term goals. The advantages of robotic-assistance in laparoscopic suturing can be hypothesized to result in more reliable mesh fixation to the rectum and apex of vagina. Recurrences have been reported to occur in 0–12.8% after RVMR and 1.5–9.7% after LVMR for ERP and in 10.4% after RVMR and 5.3–7.1% after LVMR for IRP [29, 30]. One rectal prolapse recurrence and one apical descent both occurred in the LVMR group (7.7%, 3.3% of whole cohort). However, the difference between RVMR and LVMR was not significant. The anatomical correction after RVMR and LVMR seem similar as the normal support of the posterior vaginal wall (POP-Q stage ≤ 1) was maintained in all patients in both study groups in the follow-up.

The functional results showed some deterioration within time and specifically incontinence symptoms persisted. The decrease in Wexner score (mean − 1.5 points RVMR and − 3.4 points LVMR, *p* = 0.55) is somewhat smaller than previously reported improvement after VMR, which may reflect to HRQoL results. The greater visibility and more precise dissection with robotic-assisted laparoscopy is proposed to be beneficial in preserving of autonomic nerves and thereby resulting in less postoperative constipation [2]. One non-randomized study showed better improvement in obstructed defecation symptoms after RVMR compared with LVMR [31]. In the current study the reduction seen in the obstructed defecation symptoms was also in favor of RVMR (*p* = NS). We observed an improvement in sexual function after rectopexy, which has also been reported previously [31].

The results of this study should be interpreted in the light of some limitations. Based on a large experimental survey, The European Union Seventh framework program recommended in 2013 that cost-effectiveness analyses should be expressed as costs per relevant clinical outcome, and QALY assessment for healthcare decision making should be abandoned due to methodological limitations of its use [32]. Although having an advantage for being relatively comparable across studies using same measures QALY is possibly not very responsive to disease-specific effects of intervention [32]. In concordance with previous reports of around 60–90% patients benefitting from the VMR [1, 2, 5, 13, 29–31] patients were satisfied with operative result, however, 2-year HRQoL returned to baseline. In all, the HRQoL was reduced in a wide range of dimensions in this patient group both before and after operation, when compared to age-standardized general Finnish population, which may relate to a multifactorial background (Fig. 3a, b) [33, 34]. Our results differ from the finding of a recent SF-36 HRQoL study, in which significant improvement in general health status after VMR was measured [35]. However, the symptom-specific QoL improved significantly, with PFIQ-7 combining also global pelvic floor impact. In a similar patient group of patients having an intervention for fecal incontinence, significant symptom improvement was achieved without an impact on general HRQoL [36].

The major limitation of our study is the small sample size, due to which it is underpowered to detect minor differences between the treatment groups. The study cohort may reflect the learning curve of robot operations in a single center. However, the operative outcome is comprehensively assessed by predetermined methods. The study was done using hospital perspective, i.e., not including costs and benefits for the other sectors in society. In the analysis the use of other health and social care and productivity costs between the groups were the same. It means that the societal cost-effectiveness ratio that is often used in Finland would have been very similar.

The strength of this randomized study was the comprehensive evaluation of pelvic floor dysfunction in 2-year follow-up thus providing data on clinical performance of RVMR vs. LVMR and possible explanations to the HRQoL results effecting QALY. Because there was no postoperative imaging we could not rule out the possible residual IRP, which could explain symptom recurrence [37].

## Conclusions

Robot-assisted ventral mesh rectopexy is more expensive than LVMR in the short-term, but incremental cost-effectiveness of RVMR may be acceptable at 2 and 5 years, suggesting this technique may offer value for money. The impact of RVMR and LVMR on long-term generic HRQoL is minor, despite symptomatic improvement. The reinforcement of the rectovaginal septum is similar in both techniques. More studies on this subject with larger cohorts with reasonable follow-up time are needed.
